# Tricaproin Isolated From *Simarouba glauca* Inhibits the Growth of Human Colorectal Carcinoma Cell Lines by Targeting Class-1 Histone Deacetylases

**DOI:** 10.3389/fphar.2018.00127

**Published:** 2018-03-12

**Authors:** Asha Jose, Motamari V. N. L. Chaitanya, Elango Kannan, SubbaRao V. Madhunapantula

**Affiliations:** ^1^Department of Pharmacology, JSS College of Pharmacy, JSS Academy of Higher Education and Research, Udhagamandalam, India; ^2^Department of Pharmacognosy and Phytopharmacy, JSS College of Pharmacy, Udhagamandalam, India; ^3^Department of Pharmacology, JSS College of Pharmacy, Udhagamandalam, India; ^4^Center of Excellence in Molecular Biology and Regenerative Medicine, JSS Medical College, JSS Academy of Higher Education and Research, Mysore, India

**Keywords:** *Simarouba glauca*, laxmitaru, anti-cancer activity, tricaproin, sodium butyrate, histone deacetylases, apoptosis

## Abstract

While anticancer properties of *Simarouba glauca* (SG, commonly known as Paradise tree) are well documented in ancient literature, the underlying mechanisms leading to cancer cell death begin to emerge very recently. The leaves of SG have been used as potential source of anticancer agents in traditional medicine. Recently attempts have been made to isolate anticancer agents from the leaves of SG using solvent extraction, which identified quassinoids as the molecules with tumoricidal activity. However, it is not known whether the anti-cancer potential of SG leaves is just because of quassinoids alone or any other phytochemicals also contribute for the potency of SG leaf extracts. Therefore, SG leaves were first extracted with hexane, chloroform, ethyl acetate, 70% ethanol, water and anti-cancer potential (for inhibiting colorectal cancer (CRC) cells HCT-116 and HCT-15 proliferation) determined using Sulforhodamine-B (SRB) assay. The chloroform fraction with maximal anticancer activity was further fractionated by activity-guided isolation procedure and structure of the most potent compound determined using spectral analysis. Analysis of the structural characterization data showed the presence of tricaproin (TCN). TCN inhibited CRC cells growth in a time- and dose dependent manner but not the normal cell line BEAS-2B. Mechanistically, TCN reduced oncogenic Class-I Histone deacetylases (HDACs) activity, followed by inducing apoptosis in cells. In conclusion, the anti-cancer potential of SG is in part due to the presence of TCN in the leaves.

## Introduction

*Simarouba glauca* DC *(S. glauca, S.G)*, commonly known as laxmitaru and paradise tree, belongs to Simaroubaceae family (Govindaraju et al., 2009, NGPS). Parts of *S. glauca* plant have been used extensively in traditional medicine to treat cancers (Patil and Gaikwad, 2011). For example, decoction prepared using SG leaves has been reported to be effective in treating various cancers (Rangarajan, 2003; Narendran, 2013). Supporting these traditional uses, preliminary studies by National Cancer Institute, United States demonstrated that alcoholic extracts of SG inhibited the growth of cancer cells even at a dose of 25 μg/ml.^1^ Very recently, a study by Puranik et al. (2017) showed the anti-bladder cancer activity of ethanol extract using T-24 cell line. Similarly, a separate study isolating anticancer constituents using bio activity-guided fractionation of chloroform extract of *S. glauca* twigs reported the presence of six canthin-6-one type alkaloid derivatives – (1) canthin-6-one; (2) 2-methoxycanthin-6-one; (3) 9-methoxycanthin-6-one; (4) 2-hydroxycanthin-6-one; (5) 4,5-dimethoxycanthin-6-one; and (6) 4,5-dihydroxycanthin-6-one; a limonoid, melianodiol, an acyclic squalene-type triterpenoid, 14-deacetyleurylene, two coumarins – scopoletin and fraxidin, and two triglycerides – triolein and trilinolein. Further testing found that among these molecules, only canthin-6-one, 2-hydroxycanthin-6-one, limonoid and melianodiol could inhibit the growth of human cancer cell lines (Rivero-Cruz et al., 2005). Another study isolated scopoletin, canthin-6-one, canthine-6-one dimethoxy derivatives from wood extract and showed their potential to inhibit human breast cancer cell lines MCF-7 and SK-BR-3 at 2.0 μg/ml and 5.5 μg/ml respectively (Reynertson et al., 2011). In summary, all these studies conclude that the extracts of SG contain potential anticancer agents.

Histone deacetylases (HDACs) are key enzymes involved in chromatin re-modeling and oncogenic behavior of cells (Glozak and Seto, 2007). Deregulated HDACs promote cancer cell proliferation, prevent apoptosis and increase cell migration through the modulation of histone acetylation (Marks et al., 2000). Since histone acetylation helps in the packaging of DNA, removal of acetyl groups by HDACs is likely to increase chromatin tightening, which ultimately culminate in the down-regulation of tumor suppressor genes such as p53, Bax, Bad, p21 etc. (Mariadason, 2008). Therefore strategies that inhibit oncogenic HDACs have potential to become clinically viable drugs for treating cancers wherein HDAC plays an important role in the tumor development (Mottamal et al., 2015). For instance, US FDA approved the use of suberanilohydroxamic acid (SAHA) for treating cutaneous T-cell lymphoma in the year 2006 (Mottamal et al., 2015). Likewise, Belinostat and Panobinstat were also approved by US FDA for the treatment of peripheral T-cell lymphoma and multiple myeloma (Mottamal et al., 2015). Recently, studies from our laboratory have demonstrated the potential of HDAC inhibiting benzoic acid and cinnamic acid derivatives for treating carcinomas of colon and rectum (Anantharaju et al., 2016, 2017a,b). Although many studies have demonstrated the clinical utility of HDAC inhibitors, success of these agents as monotherapies is still a major concern (Kuendgen et al., 2006; Thurn et al., 2011). Hence, search for more potent HDAC inhibitors that work alone still continues. In this regard a separate study synthesized and tested the ability of a selenium containing HDAC inhibitor, known as SelSA (Gowda et al., 2012). SelSA showed much better HDAC inhibition compared to parent compound SAHA (Gowda et al., 2012). However, further development of this compound was not considered due to its toxicity in mice at higher doses (Gowda et al., 2012).

Short and medium-chain fatty acids, and lipids extracted from various plants are the major sources of potential anticancer agents (Hamburger et al., 1987; Selvaraj, 2017). Mechanistically, fatty acids and lipids inhibit HDACs thereby retard cancer cell growth (Waldecker et al., 2008). For example, sodium butyrate helps in the treatment of cancers by inhibiting class-I HDACs thereby sensitizing cancer cells to chemotherapeutic agents as well as radiation treatment (Sealy and Chalkley, 1978; Davie, 2003; Rada-Iglesias et al., 2007). Likewise, medium chain triglycerides and fatty acids are also known to inhibit oncogenic HDACs leading to apoptosis in cancer cells (Kuefer et al., 2004). Therefore, triglycerides containing short and medium chain (4–8 carbons) fatty acids are better anti-cancer agents (Fauser et al., 2013; Narayanan et al., 2015). Hence, SG leaves were extracted with solvents of low- and medium polarity, and anti-cancer potential tested for inhibiting colorectal carcinoma (CRC) cell lines expressing elevated HDAC activity. The chloroform extract, which exhibited potent anti-cancer activity, was further fractionated and a triaceyl glyceride containing hexanoic acid (Caproic acid) purified. The purified compound identified as “Tricaproin” (TCN) using GC-MS, FT-IR, ^1^H and ^13^C NMR. The pure TCN inhibited HDAC and cancer cell growth in a dose dependent manner. Mechanistically, HDAC inhibiting TCN induced apoptosis followed by cell growth arrest. In conclusion, results of this study demonstrate the purification of HDAC inhibiting TCN from SG leaves and showed that TCN induces CRC cells growth arrest. Additional studies are currently underway to determine the safety and efficacy of TCN in animals.

## Materials and Methods

### Materials

Colorectal carcinoma cell lines HCT-116 and HCT-15 were procured from NCCS, Pune, Maharashtra, India. Human lung epithelial cell BEAS-2B was kindly provided by Dr. Rajeshkumar Thimmulappa, Associate Professor of Biochemistry, JSS Medical College. Cell culture reagents and disposables were from Life Technologies, Carlsbad, CA, United States and Tarson’s India Limited, Kolkata, India, respectively. BSA was from Hi-Media, Mumbai, Maharashtra, India. Hexanoic acid and Sulforhodamine-B were from Sigma Chemical Company, St. Louis, MO, United States. Analytical grade n-Hexane, Chloroform, Ethyl acetate, Ethanol, Silica gel 60-120 and ALUGRAM-Xtra aluminum plate SIL G/UV 254 were from Loba Chemie, Mumbai, Maharashtra, India. Pierce BCA protein estimation kit [cat#: 23227] was from Thermo Fischer Scientific, Waltham, MA, United States. Lactate dehydrogenase activity assay kit [cat#: K730–500] was from BioVision, Milpitas, CA, United States. Histone deacetylase fluorometric (HDAC) kit [cat#:10011563] from Cayman, Ann Arbor, MI, United States. Acridine orange, ethidium bromide, sodium butyrate were from Sisco Research Laboratories Pvt., Ltd., Mumbai, Maharashtra, India. Accelrys Discovery Studio 4.1 software is from Biovia, San Diego, CA, United States (Available at JSS College of Pharmacy, Udhagamandalam, Tamil Nadu, India).

### Methods

#### Preparation and Extraction of *S. glauca* Leaves

Fresh leaves from healthy, uninfected *Simarouba glauca* plant were collected from Palakkad district, Kerala, India. Plant taxonomist, Dr. N. Sasidharan, an Emeritus scientist from Kerala Forest Research Institute, Peechi, Thrissur, Kerala, India, has identified the plant. Leaves from taxonomically identified plants were processed as follows: (a) washing of leaves with tap water to remove dust and adherent materials; (b) rinsing with distilled water followed by drying under shade; (c) pulverization of completely dried leaves in to a fine powder using a sterile electric blender.

##### Sequential extraction of SG leaf powder

Hundred gram dried leaf powder was extracted successively in Soxhlet apparatus, using 500 ml Hexane (SGH), Chloroform (SGC), Ethyl acetate (SGEA), 70% aqueous Ethanol (SGE). Maceration was carried out for aqueous extraction (SGW). The solvent from each fraction was removed by evaporation at reduced pressure for 24 h; and the residue was frozen at -80°C for 48 h before lyophilization. Stock solution of each lyophilized sample was prepared by reconstituting 50mg material in 1.0 ml of 100% dimethyl sulfoxide (DMSO). The stock solutions were stored at 4°C until use.

#### Screening of Extracts for Anti-cancer Activity Using Colorectal Carcinoma Cell Lines HCT-116 and HCT-15

The anti-cancer activity of the crude samples was measured according to Madhunapantula et al. (2008). First, 5 × 10^3^ cells, representing carcinomas of colon and rectum (HCT-116, HCT-15, in 100 μl DMEM) supplemented with 10% FBS, 5% penstrep, 5% glutamax were seeded in a 96-well plate. The cells were grown in a CO_2_ incubator maintained at 37°C, 5% CO_2_ and 95% relative humidity. After about 48 h, at which point all the wells were 60–70% confluent, the cells were exposed to increasing concentration of samples (dissolved in DMSO and diluted in DMEM-10% FBS medium) for 24, 48, and 72 h. Viability of cells was measured using sulforhodamine-B assay and the percentage growth inhibition calculated by comparing with vehicle DMSO treated cells.

#### Measurement of Cell Viability Using Sulforhodamine-B Assay (SRB Assay)

Viability of cells was measured using SRB assay as described by Skehan et al. (1990). Experimentally, cells were fixed in 1/4th volume of cold 50% (w/v) TCA for 1 h at 4°C. The media was removed and the wells washed with water (200 μl × 4 times) to remove TCA and serum proteins. The plates were dried, incubated with 100 μl 0.4% SRB for 20 min to stain the cellular proteins. Next, the wells were washed quickly with 1% acetic acid (200 μl × 4 times) to remove unbound SRB. The bound SRB was solubilized in 10.0 mM Tris base solution (100 μl/well) and the absorbance measured at 490 nm in a microplate reader.

#### Isolation of Tricaproin From the Most Potent Chloroform Extract

##### Preparation of fatty acid methyl esters (FAME)

Chloroform extract (3.5 g) was subjected to Silica Gel (60–120 mesh) column chromatography using chloroform as an eluant. Based on the TLC analysis, the chloroform fractions were pooled and evaporated to obtain a dried mass of 3.1 g. This dried mixture was refluxed using a mixture of methanol and acetyl chloride (95:5, 100 ml) for 4 h, to prepare FAME, and partitioned thrice using 450 ml of n-hexane containing 0.01% butylated hydroxytoluene (BHT) (A), 400 ml of ethyl acetate (B) and 300 ml of water (C). The pooled fractions were distilled under vacuum to obtain 0.9 g (A), 0.7 g (B) and 1.2 g (C) of dried material respectively from n-hexane (A), ethyl acetate (B) and water (C) solvents.

##### Enrichment of FAME using urea crystallization method

The obtained FAME was subjected to urea complexation to separate the saturated fatty acids from the polyunsaturated fatty acids as described by Jubie et al. (2015) with slight modification. In short, the obtained FAME (0.9 g, 0.7 g, 1.2 g) was treated with methanol (9.0 ml) and hot solution of urea (3.0 g at 60°C) and the mixture was heated (60°C for 30.0 min) to get a clear solution. It was then cooled at room temperature and stored at 0°C overnight. The material was further filtered to separate the crystals settled at the bottom. The filtrates were subjected to vacuum distillation, which yielded powdered samples designated as Fraction #1 (0.32 g), Fraction #2 (0.15 g), and Fraction #3 (0.62 g) respectively from fractions A, B, and C. The pale crystals obtained (2.0 g) were further refluxed with water (20 ml) using rota vapor at 50°C under pressure. The aqueous layer thus formed was partitioned with n-hexane (1:2). The collected *n*-hexane layer was subjected to vacuum distillation, which yielded: Fraction #4 (0.49 g) and Fraction #5 (0.51 g) from A and B, respectively. Fraction C produced a turbid aqueous layer, separation of which (same as above) yielded a 0.48 g oily product (Fraction #6).

##### Characterization and structural elucidation of Tricaproin (TCN)

Fraction #6, containing more pure tricaproin (as evidenced by gas chromatography-mass spectrometry (GC-MS), was selected for structural elucidation. The infra red (IR) spectra were recorded by fixed cell method with Schimadzu 8400S Fourier transformed infra red (FT-IR) spectrophotometer with a range of 4000–400cm^-1^. The ^1^H (1–11 ppm) and ^13^C nuclear magnetic resonance (NMR) (10 – 220 ppm) spectra were recorded using Bruker Avance-II with CDCl_3_ as solvent. GC-MS analysis was carried out using Thermo trace 1300 Gas chromatogram coupled with Thermo TSQ 8000 mass spectra using XCalibur 2.0SP1 with Foundation 2.0SP1 software. The injector temperature was 250°C and the column pressure was 100 kPa. Helium was used as the carrier gas (1.0 ml/min) and the injection volume was 1.0 μL. The temperature was set at 60°C for 2.0 min and increased to 280°C (10°C/min). Mass spectrometry conditions were as follows: ion source temperature – 230°C; electron energy – 70 eV; interface temperature – 250°C; quadrupole temperature – 150°C; mass scan range – 50–500 amu; detector – MS TSQ 8000.

#### Anti-cancer Activity and Selectivity of Tricaproin

The ability of tricaproin and hexanoic acid to inhibit carcinoma cells representing colon and rectum (HCT-116 and HCT-15) and non-cancerous human bronchial epithelium cell line (BEAS-2B) was carried out as detailed before. The cells were exposed to increasing concentration (7.8–1000 μM) of TCN and HA (dissolved in DMSO and diluted in DMEM-10% FBS medium) for 24, 48, and 72 h, and IC50 values calculated using GraphPad Prism software (2009) (Prism).

#### Comparative Assessment of the Binding Potential of Tricaproin to Sodium Butyrate Binding Site of HDACs Using Discovery Studio 4.1

##### Selection of HDAC structure from Protein Data Bank

The X-ray crystallographic structures of human HDAC1 with dimeric ELM2-SANT domain of MTA 1 from the NuRd complex (PDB ID-4BKX) having 482 aminoacids, HDAC2 consisting 367 aminoacids bound with *N*-(2-aminophenyl) benzamide (PDB ID-3MAX), HDAC3 complexed with a co-repressor inositol tetraphosphate with 376 aminoacid residues (PDB ID-4A69), HDAC 8 complexed with an inhibitor complex and having 388 aminoacids (PDB ID-2V5X), and HDAC4 complexed with hydroxamic acid inhibitor benzamide having 488 amino acids (PDB ID-2VQV) were retrieved from Protein data bank. The PDB files were cleaned and the hetero-atoms (HETATM) of the receptors were removed manually (Anantharaju et al., 2017a).

##### Generation of ligand data set

The structures of ligands-Tricaproin, Hexanoic acid, Sodium butyrate were obtained from PubChem compound database^2^. “Prepare ligand” module available in Discovery studio 4.1 was used for preparation of the ligands for removing duplicates, enumerating isomers and tautomers, and generating 3D conformations.

##### Active site analysis of HDACs structure

Possible binding sites of HDACs were identified by first defining the receptor molecule using binding site tools of Discovery Studio. Active sites were selected according to PDB site records. Total active sites were evaluated for their binding to ligands, which include the control sodium butyrate, with lowest C-Dock energies and selected the best active site for the further evaluation to identify the potent HDAC inhibitor (Anantharaju et al., 2017a).

##### Molecular docking using Discovery Studio 4.1

The docking of ligands in the receptor-binding site was performed using C-docker module available in Discovery studio 4.1. C-docker docks ligands using the validated CDOCKER algorithm, a grid based molecular docking method that uses CHARMm with CDOCKER. Briefly, initial ligand conformations were sampled using high temperature molecular dynamics, which include dynamics steps (1000) and dynamic target temperature (1000) and allowed to flex during the refinement. Next, the conformations were translated into binding site and candidate poses created using random rigid-body rotations. This was subjected for simulated annealing including heating steps (2000), heating target temperature (700), cooling steps (5000) and cooling target temperature (300). A final minimization with full potential is then used to refine the ligand poses. The binding mode for all ligands to human HDACs was investigated by CDOCKER protocol (Wu et al., 2003). Different poses of protein-ligand complex were obtained after docking process with their specific CDOCKER energy and CDOCKER interaction scores displayed in output file.

#### Assessment of HDAC Inhibitory Potential of Tricaproin

Histone deacetylase (HDAC) inhibitory potential of Tricaproin and Hexanoic acid was determined using the HDAC1 nuclear extract supplied in the kit (*Ex vivo* analysis; Histone deacetylase fluorometric (HDAC) assay kit (10011563) from Cayman, USA) as well as the nuclear extract collected from the CRC cells; HCT-116 and HCT-15 upon treatment with Tricaproin and Hexanoic acid (*In vitro* analysis). Details of the procedure can be obtained from the following link: https://www.caymanchem.com/pdfs/10011563.pdf.

##### Isolation of cellular nuclei and collection of nuclear extract

Isolation of nuclear fraction was performed as described in the kit^3^. In brief, 1 × 10^6^ cells from control untreated, vehicle DMSO (1%) treated and Tricaproin and Hexanoic acid exposed flasks were collected by trypsinization, and centrifuged at 3000 rpm for 5.0 min. The pelleted cells were washed twice with PBS, and resuspended in 1.0 ml cold lysis buffer containing 10 mM Tris-HCl (pH 7.5), 10 mM NaCl, 15 mM MgCl_2_, 250 mM Sucrose, 0.5% NP-40 and 0.1 mM EGTA. After vortexing for 10.0 s the cell suspension was incubated on ice for 15.0 min, centrifuged at 900 rpm for 10.0 min at 4°C with 4.0 ml of cold sucrose cushion. The pellet collected was washed using cold 10.0 mM Tris HCl (pH 7.5) and 10.0 mM NaCl, centrifuged at 900 rpm at 4°C for 10.0 min to obtain a fraction rich in nuclei.

##### Extraction of nuclear fraction

The fraction rich in nuclei was resuspended in 100.0 μl nuclear extraction buffer made up of 50 mM HEPES-KOH (pH 7.5), 420 mM NaCl, 0.5 mM Na_2_EDTA, 0.1 mM EGTA, 10% Glycerol, and sonicated for 30.0 s. The mixture was incubated on ice for 30.0 min and centrifuged at 7000 rpm for 10 min. The supernatant containing crude nuclear extract was used for determination of HDAC activity. The protein content in the nuclear extract was estimated using BCA kit (Thermo Fischer, Waltham, MA, United States) as described below and extracts stored at -80°C until further use.

##### Estimation of total protein using BCA method

Total protein content in the nuclear extracts was determined using Pierce BCA kit from Thermo Fischer Scientific (Brown et al., 1989). A calibration graph was prepared by incubating increasing concentration of 25, 125, 250, 500, 750, 1000 and 1500 μg/ml bovine serum albumin (BSA, 10.0 μl) with 200 μl BCA reaction mixture containing of 50 parts of reagent A (made up of 0.8% sodium bicarbonate, 4% bicinchoninic acid and 0.16% sodium tartrate in 0.1 M sodium hydroxide) and 1 part of reagent B (made up of 4% cupric sulfate) at 37°C for 30.0 min, followed by measuring the absorbance at 562 nm using a multimode plate reader (PerkinElmer, Germany). Similarly suitably diluted test samples were also processed and concentration determined using the calibration curve.

##### HDAC inhibitory potential assessment using an *ex vivo* method

HDAC1 crude nuclear extract provided in the HDAC kit was used for determining the HDAC inhibitory potential of Tricaproin and Hexanoic acid. Experimentally, nuclear extracts containing 10 μg of protein is made up to 55 μl with assay buffer followed by addition of 5.0 μl fluoro-substrate to get 60 μl reaction volume. The reaction mixture was incubated for 30.0 min at 37°C. Appropriate experimental controls that include (a) no enzyme control with all components except the nuclear lysates; (b) a developer control consisting of fluorodeacetylated substrate instead of fluoro-substrate; (c) an inhibitor control with 5.0 μM of Tricostatin-A and 6, 12, 24 mM (HCT-116) and 20, 40, and 80 mM (HCT-15) sodium butyrate (a known HDAC Inhibitor); (d) TCN and CA of 75 μM (HCT-116) and 250 μM (HCT-15) and (e) a solvent control was also processed similarly. The fluorescence was read in a kinetic mode after incubating for 15.0 min at room temperature with 15.0 μl of developer. The measurements were carried out at an excitation of 350 nm and emission of 450 nm.

##### HDAC inhibitory potential assessment using *in vitro* method

*In vitro* assessment of HDAC inhibitory potential was performed as described by Senawong et al. (2013) and Anantharaju et al. (2017a). In brief, 1 × 10^6^ HCT-116 and HCT-15 cells were seeded in 10.0 ml of DMEM supplemented with 10% FBS in a 100 mm Petri plate. The exponentially growing HCT-116 and HCT-15 cells were treated with 7.8, 15.6, 31.2, 62.5, 125, and 250 μM TCN respectively for 48 h. Tricostatin A (5.0 μM), Sodium butyrate (6, 12, 24 mM for HCT-116) (20, 40, and 80 mM for HCT-15) were used as controls. The cells were trypsinized, and 10.0 μg of nuclear lysate was used for the determination of HDAC enzyme activity as detailed before.

#### Estimation of Intracellular Reactive Oxygen Species (ROS) in Colon Cancer Cells Treated With Tricaproin

Determination of ROS levels was performed according to Shailasree et al. (2015) with minor modifications (Shailasree et al., 2015; Anantharaju et al., 2017a). 0.5 × 10^4^ HCT-116 and HCT-15 cells/well were seeded in a 96 well plate and allowed to grow in a carbon dioxide incubator maintained at 5% CO_2_ and 37°C for 48 h. Then the exponentially proliferating cells were treated with 37.5, 75, 150 μM (for HCT-116) and 125, 250, 500 μM (for HCT-15) of TCN for 24 and 48 h. Traces of media in the cells were removed by washing with PBS. Then the cells were incubated with 10 μM of 2′, 7′-dichlorodihydrofluorescein diacetate (H2DCFDA, prepared in PBS) for 30.0 min and fluorescence intensity measured using a multimode plate reader operating at an excitation of 435 nm and emission of 520 nm.

#### Detection of Apoptosis by Acridine Orange and Ethidium Bromide Staining

Apoptosis detection by double staining using acridine orange and ethidium bromide was performed as detailed (Shailasree et al., 2015; Anantharaju et al., 2017a). Experimentally, 0.3 × 10^6^ HCT-116 and HCT-15 cells were seeded in 6-well plates and exposed to increasing concentrations of TCN (37.5, 75, 150 μM) and Sodium butyrate (12 mM) on HCT-116 for about 48 h. The cells were trypsinized and mixed thoroughly to obtain a single cell suspension. The trypsin was neutralized by the addition of complete medium, and 20.0 μl cell suspension incubated with a mixture containing10.0 μl of 100.0 μg/ml each of ethidium bromide and acridine orange for 5.0 min. The cells were imaged using the fluorescence microscope using TRITC and FITC filters and later merged to obtain a combined image, which exhibited green and orange cells.

#### Confirmation of Apoptosis by Measuring Caspase-3 Activity

Activation of caspase-3 and 7 is the functional end point of apoptotic cascade and is an indicator of apoptosis induction in mammalian cells (Okada et al., 2016). The caspase-3/7 fluorescence assay kit was used to measure the caspase activity^4^. Experimentally, first, 1 × 10^4^ HCT-116 and HCT-15 cells were treated with increasing concentrations of TCN at 37.5, 75, 150 μM (for HCT-116) and 125, 250, 500 μM (for HCT-15) for 48 h. Cells exposed to Sodium butyrate at 6, 12, 24 mM and 20, 40, 80 mM for HCT-116 and HCT-15 respectively served as positive control. The cells treated with 1% DMSO served as vehicle control. The plate was centrifuged at 560 rpm for 5.0 min. After aspirating the culture medium 200 μl of assay buffer was added and centrifuged at 560 rpm for 5.0 min. Hundred microliter of lysis buffer was then added and incubated on an orbital shaker at room temperature for 30.0 min followed by centrifugation at 560 rpm for 10.0 min. Ninety microliter of the supernatant from each well was then transferred to the corresponding well in a 96 well plate (black) and 10 μl of assay buffer was added. Hundred microliter of active caspase-3 positive control (1:500 in assay buffer) was added to the corresponding well. Further, the plate was incubated at 37°C for 30.0 min after adding 50 μl of substrate [combine100 μl of N-Ac-DEVD-N′-MC-R110, 400 μl DTT (1 M) assay reagent, 9.5 ml assay buffer] solution. The fluorescence intensity was measured using a multimode plate reader operating at an excitation of 485 nm and emission of 535 nm.

### Statistical Analysis

All experiments were conducted in multiple replicates and the results were expressed as mean +SEM. The results were subjected to one way ANOVA, followed by Tukey’s *post hoc* test to analyze difference between experimental and control samples. A “*P*” value of < 0.05 was considered significant. All the graphs were generated using GraphPad Prism-5 software.

## Results

### Fractionation of SG Leaves, Using Solvents, Yielded Extracts Containing Anti-cancer Agents With Good Therapeutic Index

**FIGURE 1:**
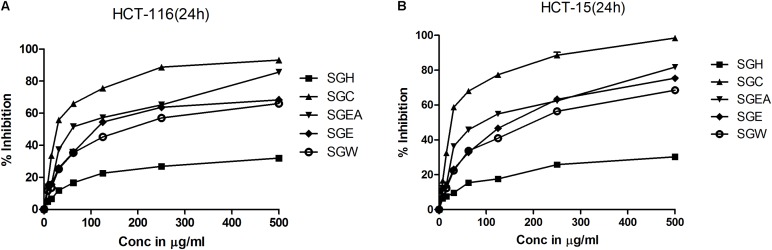
Chloroform extract of SG (SGC), compared to other extracts, more effectively inhibit the viability of colorectal carcinoma cell lines: To screen and identify the most potent extract which can inhibit the growth of colorectal cancer cell lines, first, SG leaves were sequentially extracted with solvents of increasing polarity and tested for efficacy on HCT-116 **(A)** and HCT-15 **(B)** cell lines. Analysis of the data showed a significant growth reduction with increasing concentration of the extracts at 24 h of treatment. Among the extracts tested, chloroform extract showed much better cell proliferation inhibition indicating the presence of compounds that can inhibit the growth of colorectal carcinoma cells. The screening experiments were carried out thrice with at least 4 replicate wells in each experiment. The values represented are Mean ± SEM.

To isolate the anti-cancer agents from *Simarouba glauca*, the leaves were successively extracted with n-hexane (SGH), chloroform (SGC), ethyl acetate (SGEA), hydroalcohol (70% ethanol; SGE) water (SGW) and anti-cancer potential in terms of CRC cells growth inhibition determined using SRB assay (**Figures 1A,B** and **Table 1**). Analysis of the data showed a dose- and time- dependent increase in the potency of each extract (**Figures 1A,B**, **Table 2**, and Supplementary Figures 1A–D). For instance, at 72 h of treatment of HCT-116 cells, the chloroform extract exhibited the most potent anti-cancer activity with an IC50 of ∼18.0 μg/ml compared to ethyl acetate (IC50 ∼45 μg/ml), hydroalcohol (IC50 ∼80 μg/ml) and water (IC50 ∼100.0 μg/ml) (Supplementary Figure 1B). However, the extract prepared using hexane had only minimal effect at all concentrations tested (**Figure 1A**). For example, at 500 μg/ml concentration, only about 25% growth inhibition was observed at 24 h treatment (**Figure 1A**). Extending the treatment time for 48 and 72 h improved the efficacy marginally (**Table 2** and Supplementary Figures 1A,B). Similar trend was observed upon treatment of HCT-15 cells with crude extracts (**Figure 1B** and **Table 2**). The IC50 values were found to be in the order of SGC (∼18 μg/ml), SGEA (∼43 μg/ml), SGE (∼80 μg/ml), SGW (∼115 μg/ml) (**Table 2** and Supplementary Figures 1C,D).

**Table 1:** Percentage yield of crude extracts of *Simarouba glauca* leaves.

Extractant	Abbreviation	Percentage yield (gm/100 g of dried leaves of *S.G*)
Hexane	SGH	2.08 ± 0.15
Chloroform	SGC	3.53 ± 0.10
Ethyl acetate	SGEA	2.53 ± 0.12
70% Ethanol	SGE	8.54 ± 0.10
Water	SGW	5.51 ± 0.09


*Table provides the percentage yield of *Simarouba glauca* extracts, which were prepared using solvents ranging from non-polar to polar. Extract prepared using hydro-alcohol (70% ethanol) provided maximum yield. The experiments were carried out at least three times and the values expressed as Mean + SD.*

**Table 2:** IC50 value (μg/ml) of crude extracts of *Simarouba glauca* leaves on HCT-116 and HCT-15 colorectal carcinoma cells.

Extract	HCT-116			HCT-15		
		
	24 h	48 h	72h	24 h	48 h	72 h
SGH	ND^1^	ND	ND	ND	ND	ND
SGC	30.98 ± 0.33	23.63 ± 0.37	18.08 ± 0.17	29.37 ± 0.12	23.66 ± 0.64	18.15 ± 0.11
SGEA	78.86 ± 1.94	49.65 ± 1.96	45.84 ± 1.10	92.29 ± 2.05	46.87 ± 1.36	42.74 ± 0.79
SGE	129.67 ± 0.91	116.30 ± 3.20	82.27 ± 4.23	137.06 ± 3.23	117.46 ± 3.78	79.55 ± 1.65
SGW	169.16 ± 0.96	123.00 ± 4.47	98.69 ± 4.31	179.20 ± 3.29	129.13 ± 1.73	114.86 ± 2.78


*Table represents the IC50 values of crude extracts on human colorectal carcinoma cell lines HCT-116 and HCT-15 at 24, 48, and 72h. Dose range of 7 to 500 μg/ml was tested and IC50 values were calculated. The chloroform extract showed a potent anti-colorectal cancer activity with an IC50 of 18 μg/ml at 72 h. These experiments were carried out three times with at least 4 replicate wells in each experiment. The values are expressed in Mean ± SEM. ND, not determined.*

**FIGURE 2:**
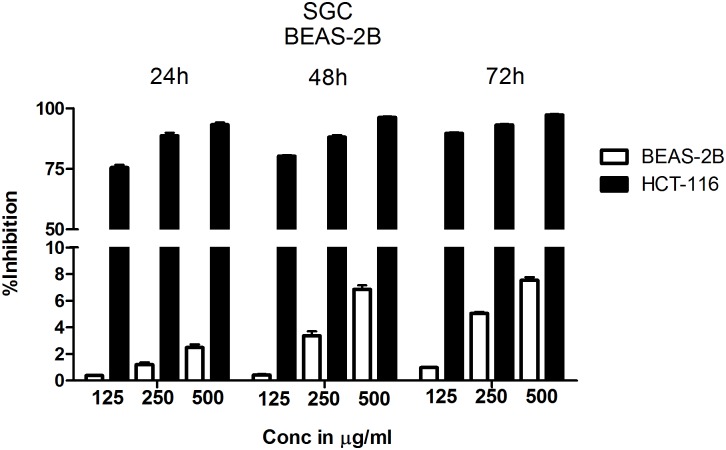
Chloroform extract is selective to colorectal carcinoma cell line HCT-116 compared to normal lung epithelial cell line BEAS-2B. To determine the therapeutic index of chloroform extract, normal lung epithelial cell line BEAS-2B and colorectal carcinoma cell line HCT-116 were treated with increasing concentration of the extract for 24, 48, and 72 h. The number of viable cells determined using SRB and percentage inhibition calculated. Analysis of the data showed a significant difference in the efficacy of chloroform extract with HCT-116 cells more responsive to treatment compared to BEAS-2B, indicating the selectivity of chloroform fraction. The data shown is the Mean ± SEM of three independent experiments with 4 replicate wells in each experiment.

Since chloroform extract showed better potency compared to other extracts, next, the selectivity was determined by comparing the percentage growth inhibition of normal bronchial epithelial cell line BEAS-2B with CRC cell line HCT-116 at 125 μg/ml, 250 μg/ml and 500 μg/ml concentrations treated for 24, 48, and 72 h. The chloroform extract showed much better selectivity to HCT-116 cell line compared to BEAS-2B cells (**Figure 2**). The selectivity is much better at lower concentrations compared to higher concentration (**Figure 2**). For example, at 24 h treatment with 125 μg/ml chloroform extract, only 0.38% growth inhibition was observed in BEAS-2B cells compared to 75% in case of HCT-116. However at 500 μg/ml concentration the % inhibition of BEAS-2B and HCT116 cells increased to ∼2% and ∼95% respectively (**Figure 2**). The selectivity of extract toward cancer cells decreased as the incubation time increases from 24 to 72 h, which is primarily due to increased growth retardation of even normal cells (**Figure 2**).

### Purification and Characterization of Anti-cancer Agent Tricaproin From Chloroform Extract

**FIGURE 3:**
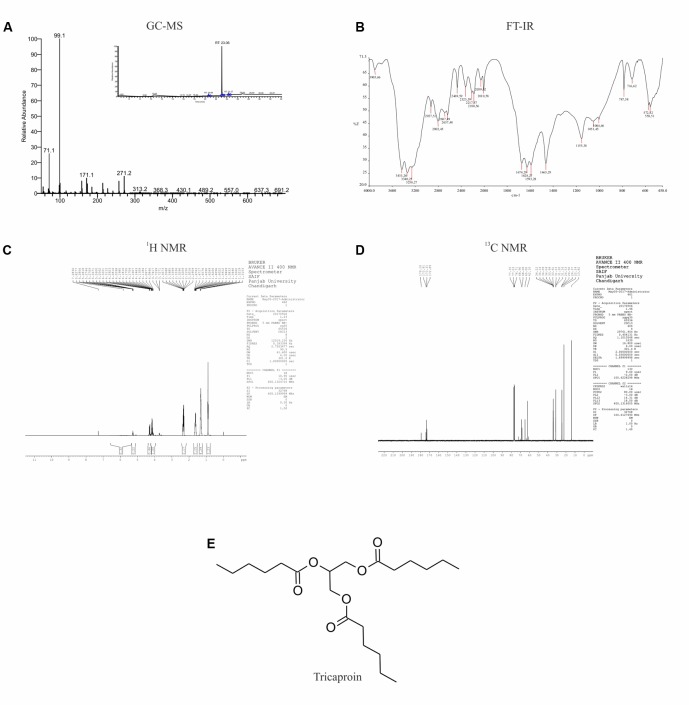
Elucidation of the structure of purified compound: The pure compound obtained by the fractionation of chloroform extract was subjected for structural characterization using **(A)** GC-MS; **(B)** FT-IR; **(C)** ^1^H-NMR; and **(D)** ^13^C-NMR. Analysis of the data showed the presence of tricaproin **(E)**. Tricaproin is a triglyceride made up of 3 hexanoic (caproic) acid residues ester linked to the hydroxylic groups of glycerol.

Results of anti-cancer agent screening and selectivity data suggested the presence of potent anticancer agents with good safety profile in chloroform extract. Hence, the chloroform extract was further fractionated using silica gel column chromatography followed by preparation of fatty acid methyl esters as detailed in materials and methods (Supplementary Figure 2). This fractionation procedure yielded 6 fractions (Supplementary Table 1 and Supplementary Figure 3). Analysis of all these fractions by GC-MS (Electron ionization-Time of Flight (EI-TOF) showed that Fraction-6 with a yield of 0.24 g is pure compared to Fractions-1 to -5 (**Figure 3A** and Supplementary Tables 2–5). The fragment ions obtained (%intensity) are – 271 (20), 215 (5), 171 (5), 159 (10), 99 (100), 71 (50). Comparison of these fragment-ions with the mass spectral database suggested the presence of tricaproin (C_21_H_38_O_6_) with a calculated molecular mass 386.529 g/mol. Next, Fraction-6 was subjected for further structural characterization using FT-IR (fixed cell method), ^1^H NMR and ^13^C NMR (**Figures 3B–D**). Analysis using FT-IR showed characteristic wavelength frequencies representing C-H stretching (2927 cm^-1^), C-H bending (1465 cm^-1^), C = O stretching (1674 cm^-1^), C-O stretching (1155 and 1051 cm^-1^) (**Figure 3B**). Analysis using 400 MHz ^1^H NMR yielded additional structural details confirming the presence of - CH(COO)_3_ (δ 5.5 s, 1H), -CH_2_COO (δ 2.5–2.25 m, 6H), (CH_2_)_3_ (δ 1.70–1.60 m, 6H), -(CH_2_-CH_2_)_3_ (1.50–1.20 m, 12H) (**Figure 3C**). Subsequent analysis using ^13^C NMR (in CDCl_3_-d6) yielded the following structural details: (a) presence of >C = O ester (173.91, 173.31, 172.85 δ ppm); (b) >C-O, -C-O- bonds 68.28, 65.00, 62.10 (δ ppm); (c) -C-C = O ester (34.14, 34.09, 34.04 δ ppm); (d) -CH_2_ (33.99, 33.91, 31.23, 31.20, 31.16, 24.53, 24.50, 24.37, 22.24 δ ppm), and (e) -CH_3_ (13.82 δ ppm) (**Figure 3D**). Taken together, these structural details confirmed the presence of a tricaproin in the pure fraction, which showed good anticancer activity (**Figure 3E**).

### Tricaproin and Hexanoic Acid (Caproic Acid) Inhibit Colorectal Carcinoma Cells Growth *in Vitro*

**FIGURE 4:**
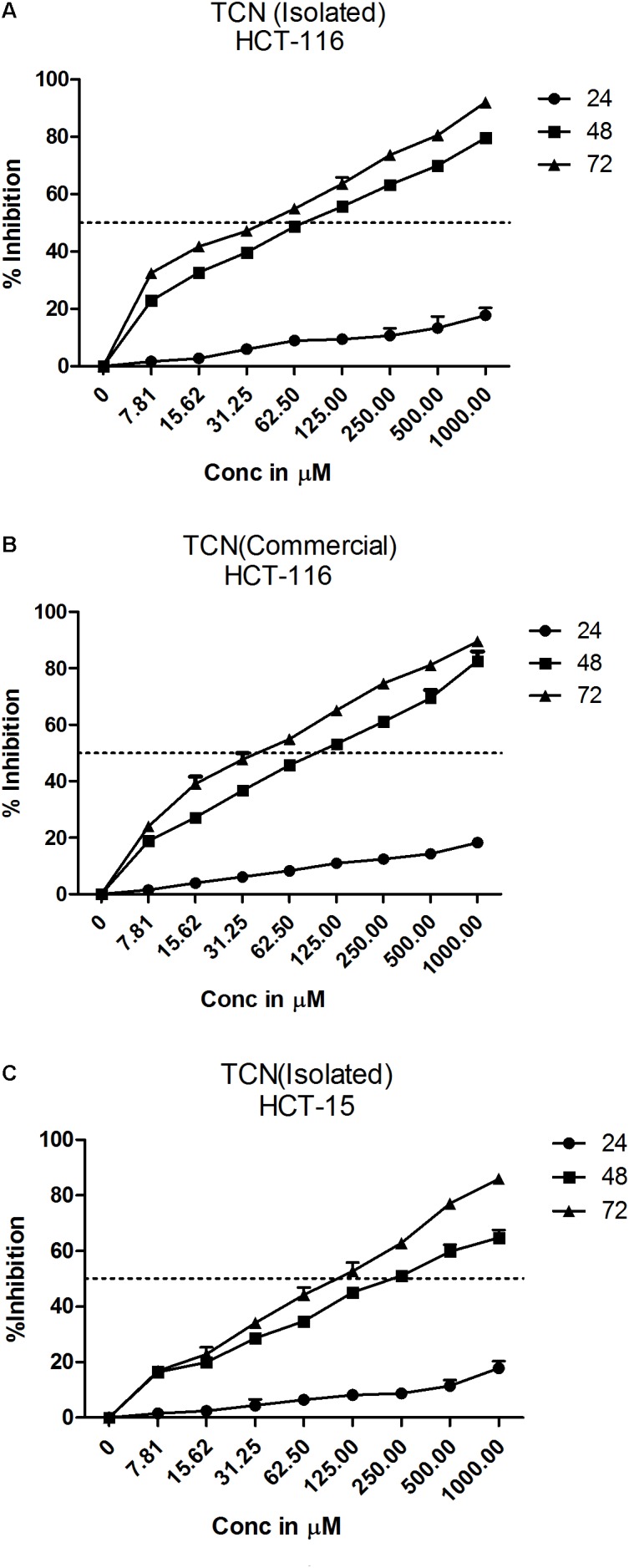
Comparative assessment of commercially available tricaproin with that of the tricaproin purified from SG leaf. In order to determine whether the TCN purified from SG leaves has similar cytotoxic properties compared to commercially available TCN, colorectal carcinoma cells HCT-116 **(A,B)** and HCT-15 **(C)** were treated with increasing concentrations of TCN isolated (**A**: HCT-116 cell line and **C**: HCT-15 cell line) as well as TCN obtained from commercial sources for 24, 48, and 72 h. Percentage growth inhibition was calculated and the data compared between the two TCN molecules. Analysis of the data showed no much difference between the TCN isolated from SG leaves and the TCN obtained from commercial vendors. The data represented is the Mean ± SEM of three independent experiments.

Tricaproin is a molecule produced by the condensation of each of the hydroxylic groups of glycerol with hexanoic acid (also known as caproic acid) (**Figure 3E**). In order to determine and further confirm functionally that the Fraction-6 contain tricaproin, the cytotoxic potency of the commercially available tricaproin (procured from Sigma, St. Louis, MO, United States) was compared with the purified Fraction-6 on HCT-116 cells (**Figures 4A,B**). Analysis of the data showed similar growth inhibitory properties when tested on HCT-116 (**Figures 4A,B**). TCN was also tested on HCT-15 cell line, which yielded a dose and time dependent response (**Figure 4C**). Next, to check whether the monomeric hexanoic acid and glycerol exhibit anti-cancer activity, HCT-116 and HCT-15 cells were treated with increasing concentration of hexanoic acid and glycerol and viability determined using SRB at 24, 48, and 72 h (Supplementary Figures 4A–D). The data showed a significant growth reduction, which is similar to parent tricaproin, only with hexanoic acid treatment (Supplementary Figures 4A,B) but not with glycerol exposure (Supplementary Figures 4C,D). Therefore, the active molecule in tricaproin is hexanoic acid but not glycerol.

Selectivity of the TCN was tested against normal bronchial epithelial cell line BEAS-2B and CRC cell line HCT-116 at 250, 500, and 1000 μM concentrations treated for 24, 48, and 72 h. The compound exhibited more selectivity toward HCT-116 cell line compared to BEAS-2B cells (Supplementary Figure 5). For example, at 48 h treatment with 125 μg/ml TCN, only 1.4% growth inhibition was seen in BEAS-2B cells compared to 64% in case of HCT-116 cells (Supplementary Figure 5). Supporting the TCN cytotoxicity on cancer cell lines, analysis of lactate dehydrogenase released in to the cell culture media, an indicator of cell death, also showed that compound treatment induced death in colorectal cancer cells (Supplementary Figure 6).

### Tricaproin and Hexanoic Acid Inhibit HDAC Activity More Potently Than Sodium Butyrate

**FIGURE 5:**
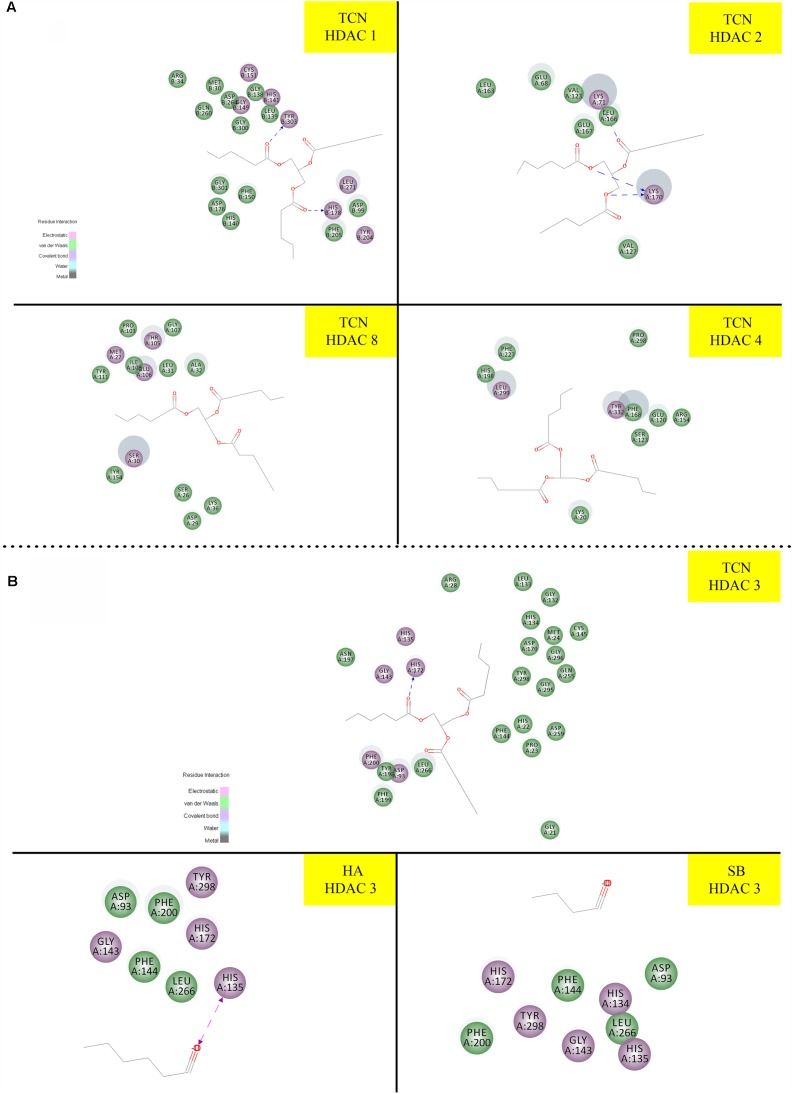
Molecular docking studies demonstrating the ability of TCN to bind to Class-I HDACs: Since sodium butyrate is a known inhibitor of HDAC proteins, we have tested the ability of TCN to bind to sodium butyrate binding site of HDAC using Discovery software. As shown TCN binds to Class I HDACs 1, 2, 8 and Class II HDAC-4 **(A)**. Further analysis showed that TCN exhibited much better binding to HDAC-3 **(B)**. However, hexanoic acid and sodium butyrate exhibited poor binding to HDAC3 **(B)** indicating the selectivity of TCN toward HDAC3.

Sodium butyrate (SB) is a known inhibitor of HDAC. Structurally, SB is shorter by two -CH_2_ groups to hexanoic acid (Supplementary Figure 7). Since SB is known to inhibit HDAC activity, we have hypothesized that the hexanoic acid and tricaproin might also inhibit HDAC activity. To test this hypothesis, tricaproin, hexanoic acid and SB were first docked in to the sodium butyrate binding site of Class-I (HDACs -1, -2, -3, and -8) and Class-II HDAC (i.e., HDAC-4) and the binding energies compared (**Table 3** and **Figures 5A,B**). Analysis of the data showed a significant strong binding of TCN especially on HDAC 3 (**Figure 5B**), as evidenced by higher negative C-Docker energy as well as C-Docker interaction strength, compared to SB or Hexanoic acid to all HDACs evaluated (**Table 3**). The order of binding of TCN to Class-I HDACs is HDAC-3 > HDAC-2 > HDAC-1 > HDAC-8. Class-II HDAC, i.e., HDAC-4 showed the lowest C-Docker energy (**Table 3**). Therefore, *in silico* analysis showed better binding of TCN to Class-I HDACs compared to Class-II HDAC.

**Table 3:** Comparison of HDAC binding energies and key amino acid residues involved in the interactions of Tricaproin, Hexanoic acid, and Sodium butyrate.

Target	Compound	C Docker energy (Kcal/mol)	C Docker Interaction Energy (Kcal/mol)	Interacting residues
HDAC 1	SB	-24.701	-23.159	HIS 178, TYR 303, ASP 176, MET 30, LEU 139, CYS 151
	TCN	-52.499	-43.844	HIS 178, MET 30, LEU 139, CYS 151
	HA	-19.574	-19.992	TYR 303, MET 30, LEU 139, CYS 151
HDAC 2	SB	-25.158	-23.870	LYS (71 and 170), LEU 166
	TCN	-56.974	-45.540	LYS (71 and 170), LEU 166
	HA	-30.291	-29.548	LYS (71 and 170), LEU 169
HDAC 3	SB	-18.129	-17.151	HIS (135, 172), TYR 298, PHE 144, PHE 200
	TCN	-62.206	-55.954	HIS (134, 135, 172), GLY 143, CYS 145, LEU 133, PRO 23
	HA	-22.797	-20.664	HIS (134, 135, 172)
HDAC 8	SB	-16.799	-16.452	THR 105, TYR 154, ILE 108
	TCN	-49.471	-38.570	THR 105, GLU 106, SER 30, ALA 32, LEU 31
	HA	-8.049	-2.252	TRP 141, ILE 115, LEU 155
HDAC 4	SB	-40.117	-39.182	LYS 20, ARG (37 and 154), PHE 168
	TCN	-43.016	-30.362	LEU 299, HIS 198, PHE 227
	HA	-43.189	-41.172	LYS 20, ARG (154 and 37), PHE 168, TYR 332


*Table: Comparative *in silico* analysis of the binding strengths of TCN to Class-I HDACs and Class-II HDAC.*

**FIGURE 6:**
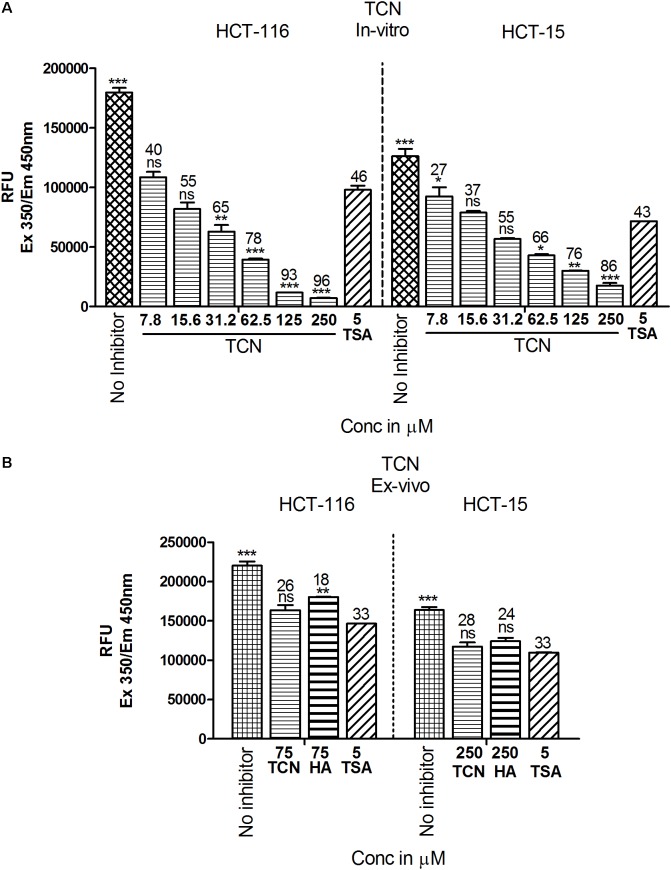
TCN inhibited HDAC activity *in vitro*: Based on the *in silico* data, it is predicted that TCN binds to Class-I HDACs with more selectivity to HDAC3, which is likely the factor responsible for HDAC activity inhibition and cell growth retardation. To test whether this hypothesis, colorectal carcinoma cells were incubated with increasing concentration of TCN and nuclear and cytosolic fractions collected. HDAC activity compared to vehicle treated cells was assessed using a kit. Analysis of the data showed that TCN, indeed, inhibited HDAC activity *in vitro* when tested against HCT-116 and HCT-15 cells **(A)**. To further test the efficacy *ex vivo*, the nuclear extracts were isolated from HCT-116 and HCT-15 cells and treated with TCN at 75 and 250 μM concentration and percentage inhibition of HDAC activity calculated. HA and TSA were used as controls **(B)**. HDAC inhibition experiments were performed thrice with 3 replicates in each experiment and values expressed in Mean ± SEM.

Since the *in silico* data showed better binding of TCN to Class-I HDACs, next, the ability of TCN to inhibit HDAC activity was determined by exposing HCT-116 and HCT-15 cells, which are known to contain elevated HDACs, to increasing concentrations of TCN for 48 h. Nuclear extract from the cells not exposed to (untreated), or exposed to TCN or control TSA was isolated and HDAC activity measured as detailed in methods section (Senawong et al., 2013). A significant dose dependent decrease in HDAC activity was observed upon treatment of HCT-116 and HCT-15 cells (**Figure 6**). At 125.0 μM concentration, about 93 and 76% inhibition was observed, respectively, for HDACs isolated from HCT-116 and HCT-15 cell lines, confirming the *in silico* observations (**Figure 6A**). However, interestingly, when the TCN, HA and positive control TSA were incubated with nuclear extract collected from HCT-116 and HCT-15 cell lines, the percentage inhibition was significantly low (86% vs. 28% at 250 μM on HCT-15 cells, **Figure 6B**) compared to the *in vitro* experiments wherein cells were first exposed to the compounds, followed by assessing the HDAC activity in the nuclear extract (**Figure 6A**). To further test this interesting observation, HDAC1 (supplied as a control in HDAC kit) was incubated with 250 μM TCN, 250 μM Hexanoic acid and 5.0 μM TSA and percentage inhibition compared to control vehicle exposed HDAC1 calculated. A non-significant 12 and 8% inhibition was observed, respectively with TCN and HA treatments (Supplementary Figure 8A). Even the positive controls TSA and SB exhibited similar reduction in percentage HDAC activity inhibition when tested using isolated nuclear extract or commercial HDAC1 (Supplementary Figures 8A–C)

### Tricaproin Induced Reactive Oxygen Species in Colorectal Carcinoma Cells

**FIGURE 7:**
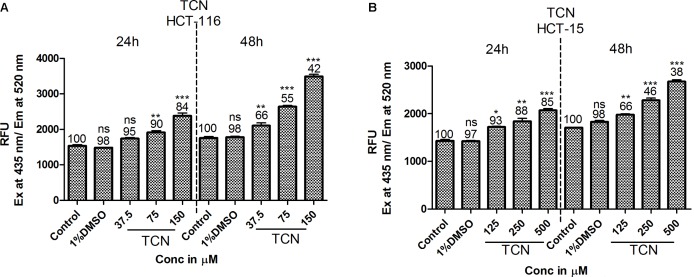
HDAC inhibitor TCN enhances cellular ROS: HDAC inhibitors are known for their ability to induce cellular ROS. Hence, the ability of TCN to induce cellular ROS was measured using H2DCFDA procedure as detailed in materials and methods. As predicted, TCN induced cellular ROS thereby decreased the number of viable cells, especially at 48 h treatment on HCT-116 **(A)** and HCT-15 **(B)** cells. These experiments were carried out at least 3 times to check the reproducibility and the results expressed as Mean ± SEM.

HDAC is a known regulator of antioxidant enzymes super oxide dismutase (SOD), peroxidase and catalase in cells (Espinosa-Diez et al., 2015). Hence, accumulation of reactive oxygen species (ROS) is a characteristic features of HDAC inhibitors (Rosato et al., 2008). Furthermore, prior studies have demonstrated that HDAC inhibitors such as SAHA, SelSA, SB, benzoic acid and cinnamic acid derivatives induce cancer cells death by increasing the levels of ROS (Ruefli et al., 2001; Sanmartín et al., 2012; Anantharaju et al., 2017a; Salimi et al., 2017). Since, tricaproin also exhibited HDAC inhibitory activity, it is predicted that induction of CRC cells death due to tricaproin treatment might be due to elevated ROS levels. Therefore, to test this hypothesis, HCT-116 and HCT-15 cells were treated with increasing concentration of TCN and levels of ROS estimated as detailed in methods. As predicted, treatment of HCT-116 and HCT-15 cells with increasing concentrations of TCN significantly increased the reactive oxygen species at 24 and 48 h (**Figures 7A,B**). An about twofold increase in ROS was observed at 150.0 μM concentration in HCT-116 cell line at 48 h of treatment (**Figure 7A**). Similarly an about 1.6-fold increase in ROS was observed upon treatment of HCT-15 cells with 500.0 μM tricaproin (**Figure 7B**). The increase in ROS correlated directly with decrease in cell viability (Numbers on top of each bar).

### Tricaproin Induced Apoptosis in Colorectal Carcinoma Cells

Increased ROS in a cell induces apoptosis thereby promote cell death (Simon et al., 2000). In order to determine whether TCN-treatment induced ROS also triggered apoptosis in HCT-116 and HCT-15 cells, the cells were stained with acridine orange and ethidium bromide, and the levels of caspase-3 (a marker for apoptosis) measured as detailed in materials and methods.

**FIGURE 8:**
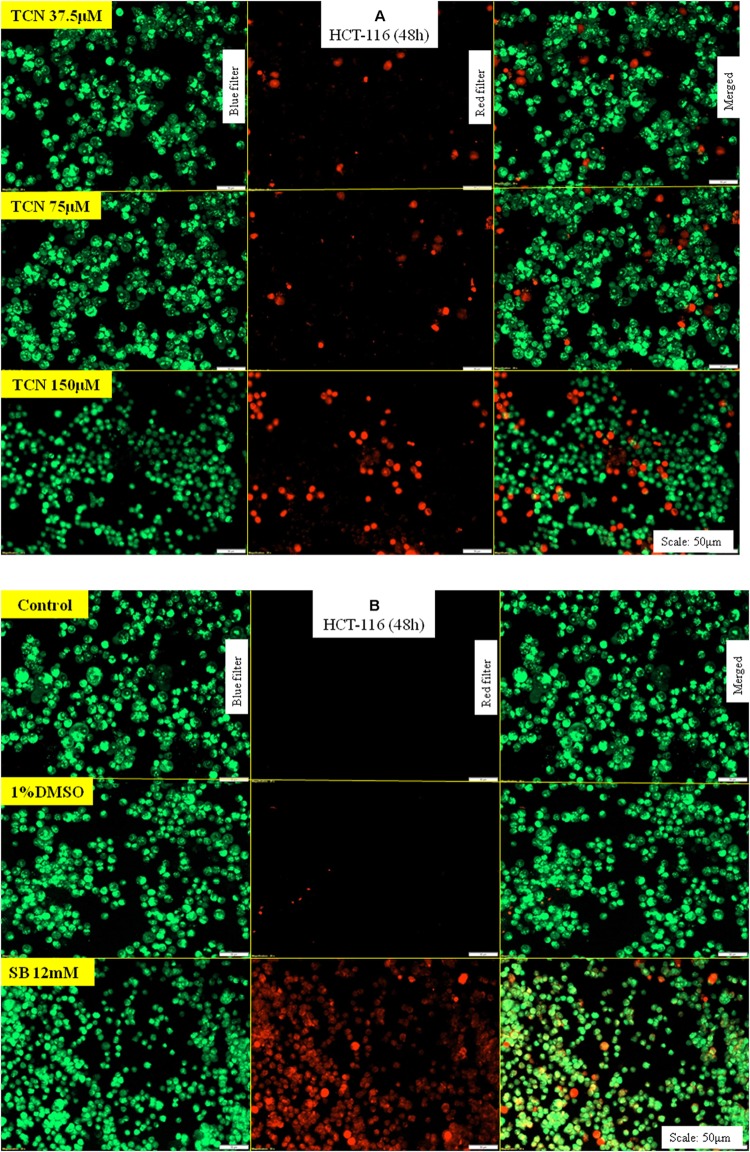
TCN triggered apoptosis in HCT-116 cells: Acridine orange and ethidium bromide staining procedure helps to differentiate proliferating and apoptotic cells using a fluorescent microscope at a magnification of 20X. Since TCN inhibited cancer cells growth, now, the mechanistic basis for cell growth inhibition was assessed. As shown, treatment of colon cancer cells HCT-116 with TCN induced apoptosis as evidenced by the presence of condensed chromatin (orange stained cells) **(A)**. Untreated and vehicle treated cells exhibited no signs of apoptosis as there were no orange stained cells in these fields. The weak HDAC inhibitor sodium butyrate also induced apoptosis, but at 12 mM concentration **(B)**.

First, TCN treated and untreated HCT-116 cells (48 h) were stained with ethidium bromide and acridine orange to evaluate apoptosis. Theoretically, the cells undergoing apoptosis exhibit condensed chromatin and show orange-red color compared with the live cells that appear green when observed under fluorescence microscope. Analysis of photomicrographs showed significantly high orange and red stained cells when treated with TCN (**Figure 8A**). However, no such stained cells were observed in untreated or vehicle 1% DMSO treated cells (**Figure 8B**). The number of orange stained cells increased with increasing TCN concentration.

**FIGURE 9:**
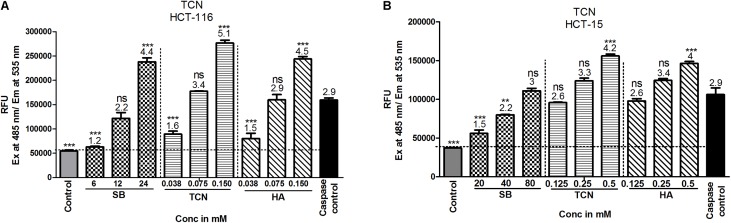
TCN induced cell death is mediated by the induction of caspase-3 activity *in vitro*: Induction of caspases is one of the mechanisms often reported for anti-cancer agents. Therefore, to check whether TCN also triggers caspase-3 activity, HCT-116 **(A)** and HCT-15 **(B)** cells were treated with increasing concentrations of TCN for 48 h and cell lysates collected as detailed in materials and methods. The data showed induction of caspase-3 activity with increasing TCN concentration in both cell lines. The component hexanoic acid also induced apoptosis in these cell lines in a dose dependent fashion indicating that the effect could be due to short chain fatty acid hexanoic acid. Pure caspase-3, supplied in the kit, was used as positive control in this study. These experiments were carried out 3-times with 4 replicates in each experiment. The data represented is the Mean ± SEM.

Induction of caspase-3/7 mediated apoptosis is a characteristic feature of HDAC inhibitors (Jasek et al., 2012; Okada et al., 2016). Therefore, the levels of caspase-3/7 were estimated using a fluorimetric Caspase-3/7 assay kit as detailed in materials and methods. A dose dependent increase in caspase-3 activity was observed upon exposing HCT-116 and HCT-15 cells to TCN as well as control compounds SB and hexanoic acid (**Figures 9A,B**). The efficacy of TCN and hexanoic acid for inducing caspase-3 activity is much higher compared to SB in both the cell lines (**Figure 9**). For example, at 0.038 mM concentration an about 1.6 and 1.5 fold increase in caspase-3 activity compared to control DMSO treated cells was observed respectively with TCN and hexanoic acid treatment. However, even at 6.0 mM concentration of SB, only 1.2 fold increases in caspase-3 activity was observed. Similar trend was observed even in HCT-15 cell line (**Figures 9A,B**)

## Discussion

Evidences from traditional medicine reports and recent success with naturally occurring anti-cancer agents stimulated the scientific community to screen and identify potential phytochemicals for treating cancers (Shoeb, 2006). In addition, the advantages with natural products such as low-toxicity and easy availability also provided additional strength to anti-cancer agents screening research (Cragg and Newman, 2005; Shah et al., 2013). *Simarouba glauca*, a Simaroubaceae family plant, is a rich source of bitter substances with potential anti-cancer properties (Patil and Gaikwad, 2011). To date, not much is known about their anti-cancer potential. Moreover, it is also not known whether any other type of compounds (other than bitter substances) do present in SG leaf extracts (Puranik et al., 2017). Therefore, in this study, leaves of *Simarouba glauca* were extracted with solvents of increasing polarity and anti-cancer activity screen performed, which identified chloroform extract as a better source for compounds with potent anti-cancer activity (Rivero-Cruz et al., 2005; Puranik et al., 2017). Chloroform alone or in association with methanol (2:1) has been widely used to extract short chain fatty acids and triglyceride esters (King and Min, 1998). The chloroform extracted compound was identified as tricaproin (TCN) by structural analysis using GC-MS, FT-IR and ^1^H and ^13^C NMR (NIST, da Rocha et al., 2007, SDBS). TCN is an ester made up of three hexanoic acids ester linked to glycerol (da Rocha et al., 2007). Once enter in to cell, the tricaproin undergo hydrolysis to produce hexanoic acid and glycerol. Since short chain fatty acids such as butyrate (a weak inhibitor of HDAC) are known to inhibit HDAC, the ability of tricaproin and hexanoic acid were tested for their potency to reduce HDAC activity (Kuefer et al., 2004; Nande and Claudio, 2014). As predicted, TCN and hexanoic acid inhibited the HDAC activity and reduced cancer cell proliferation rates significantly compared to sodium butyrate. Further analysis showed that the glycerol moiety of tricaproin is not effective in reducing cell proliferation and HDAC activity, hence, the active component causing HDAC inhibition and cell death is hexanoic acid. Since, TCN is made up of 3 molecules of hexanoic acid, TCN is a preferred choice for delivering the active molecules compared to hexanoic acid as such.

Inhibition of HDAC is one of the viable strategies for inhibiting cancer cells growth (Glozak and Seto, 2007). Several *in vitro* and *in vivo* studies as well as clinical trials using HDAC inhibitors have demonstrated the potential use of these pharmacological agents for cancer therapy (Mottamal et al., 2015). Moreover, class-I HDACs have been shown to be over expressed in several carcinomas including colorectal cancers (Mariadason, 2008). Elevated HDACs promote cell proliferation, inhibit apoptosis and induce drug resistance in cancers (Glozak and Seto, 2007). Targeted inhibition of HDAC using naturally occurring pharmacological agents such as benzoic acid and cinnamic acid derivatives, and synthetic SelSA were demonstrated to retard CRC cells *in vitro* and in animals (Ruefli et al., 2001; Sanmartín et al., 2012; Gowda et al., 2012; Anantharaju et al., 2017a; Salimi et al., 2017). Whereas some of these inhibitors found successful in clinical trials for inhibiting AML and CML, other compounds have not shown promising results either due to poor efficacy or due to systemic toxicity (Mottamal et al., 2015). Therefore, several research groups continued their efforts to screen and identify HDAC inhibitors from natural sources (Anantharaju et al., 2017a). Tricaproin is one such naturally occurring inhibitor of HDAC. Mechanistically, tricaproin inhibited class-I HDACs, thereby increased the levels of apoptosis mediated by caspase-3. However, it is currently unknown whether any other proteins do have a role in the apoptosis induction. Several reports have shown the upregulation of p53, Bax and Bad upon HDAC inhibition (Richon et al., 2000; Sawa et al., 2001). Therefore, in the subsequent studies the effect of inhibiting HDAC using TCN will be evaluated further to assess the expression of tumor suppressor as well as oncogenic molecules. In addition, the future studies should focus on testing the efficacy of TCN for inhibiting tumor growth as well as metastasis in experimental animal models.

## Conclusion

Results of our study report the purification and characterization of a Class-I HDAC inhibitor, tricaproin, from *Simarouba glauca*. Unlike sodium butyrate, a weak inhibitor of HDAC, TCN inhibited HDAC activity with more potency, hence, could be considered for further development.

Although the study showed thorough characterization of TCN, and initial testing of purified TCN in cell based assays, currently the study suffers from lack of in depth mechanistic based experiments. In summary, this is the first report demonstrating the purification and characterization of a lipid based HDAC inhibitor from *Simarouba glauca*

## Authors Note

This article is dedicated to Prof. M. N. Satishkumar, Professor of Pharmacology, JSS College of Pharmacy, Udhagamandalam, Tamil Nadu, India who breathed his last on 17th March 2015 after fighting against colorectal carcinoma.

## Author Contributions

AJ has performed the experiments, compiled the data, prepared tables and graphs. MC has performed the isolation and purification. EK and SM have assisted in designing, drafting, and editorial corrections of the manuscript.

## Conflict of Interest Statement

The authors declare that the research was conducted in the absence of any commercial or financial relationships that could be construed as a potential conflict of interest.

## Footnotes

1 http://www.revalgo.com/blog/category/simarouba-glauca/

2 https://pubchem.ncbi.nlm.nih.gov/

3 https://www.caymanchem.com/pdfs/10011563.pdf

4 https://www.caymanchem.com/product/10009135
