# Supplementary material for: Tricaproin Isolated From Simarouba glauca Inhibits the Growth of Human Colorectal Carcinoma Cell Lines by Targeting Class-1 Histone Deacetylases
Source: Front Pharmacol. 2018 Mar 12;9:127. doi: 10.3389/fphar.2018.00127 (PMC5857563; doi:10.3389/fphar.2018.00127)
Supplement: Supplementary file 1 [file Table_1.DOC]

***Supplementary Table 1: Tentative identification of compounds from chloroform extract of*** Simarouba glauca

| **Fraction** | **Retention time (Minutes)** | **Compounds identified by library search** |
| --- | --- | --- |
| F1 | 23.47  24.37  26.65  28.69 | 9,12,15-Octadecatrienoic acid  Phthalic acid, di(2-propylpentyl) ester  13-Docosenamide,(Z)  (22S)-21-Acetoxy-6à,11á-dihydroxy-16à,  17à-propylmethylenedioxypregna-1,4-diene-3,20-dione |
| F2 | 20.71  24.37  26.73 | 6,9,12,15-Docosatetraenoic acid methyl ester  Phthalic acid, di(2-propylpentyl)ester  13-Docosenamide,(Z) |
| F3 | 19.22 | Phthalic acid, butyl hept-3-yl ester |
| F4 | 19.03  20.71  20.95 | Hexadecanoic acid methyl ester  Octadecanoic acid, 4-hydroxy methyl ester  Methyl stearate |
| F5 | 19.04  23.08 | Hexadecanoic acid methyl ester  Tricaproin |
| F6 | 23.06 | Tricaproin |

**Supplementary Table 2: Comparison of** FT-IR spectra of Tricaproin

| Functional group | Wavelength | |
| --- | --- | --- |
|  | Reported | Isolated molecule |
| C-H stretching  C-H bending  C=O stretching  C-O stretching | 2958  1435  1743  1100 | 2927  1465  1674  1155,1051 |

**Supplementary Table 3: Comparison of GC-MS fragment ions** of Tricaproin

|  | m/z (relative intensity %) | |
| --- | --- | --- |
| Chemical moiety | Reported | Isolated |
| C_21_H_38_O_64_  C_15_H_27_O_4_  C_11_H_19_O_4_  C_9_H_16_O_3_  C_6_H_11_O  C_5_H_11_  C_3_H_7_ | 386 (0)  271(10)  214 (5)  171 (10)  99 (100)  71(22)  43 (22) | 386 (0)  271(12)  214 (6)  171 (10)  99 (100)  71(26)  43(not shown) |

**Supplementary Table 4: Comparison of ^1^**H-NMR of Tricaproin

|  | Signal | |
| --- | --- | --- |
| Chemical moiety | Reported | Isolated |
| s, 1H, CH(COO)_3_  m, 4H, CH_2_(OCO)_2_  m, 6H, >CH_2_COO  m, 6H, -(CH_2_)_3_  m, 12H, -(CH_2_-CH_2_)_3_- | 5.27  4.30  2.32  1.62  1.30-0.89 | 5.5  4.32  2.5-2.25  1.70-1.60  1.50-1.20 |

**Supplementary Table 5: Comparison of ^13^**C-NMR of Tricaproin

|  | Signal | |
| --- | --- | --- |
| Chemical moiety | Reported | Isolated |
| >C=O ester  >C-O, -C-O-  -C-C=O, ester  -CH_2_  -CH_3_ | 173.24, 173.24, 172.83  69.04, 62.18,62.18  34.22, 34.06,34.06  31.30, 31.30, 31.30, 24.60, 24.60, 24.60, 22.32,22.32  13.87 | 173.91, 173.31, 172.85  68.28, 65.00, 62.10  34.14, 34.09, 34.04  33.99, 33.91, 31.23, 31.20, 31.16, 24.53, 24.50, 24.37, 22.24  13.82 |
