# Supplementary material for: Tricaproin Isolated From Simarouba glauca Inhibits the Growth of Human Colorectal Carcinoma Cell Lines by Targeting Class-1 Histone Deacetylases
Source: Front Pharmacol. 2018 Mar 12;9:127. doi: 10.3389/fphar.2018.00127 (PMC5857563; doi:10.3389/fphar.2018.00127)
Supplement: Supplementary file 2 [file Data_Sheet_1.DOC]

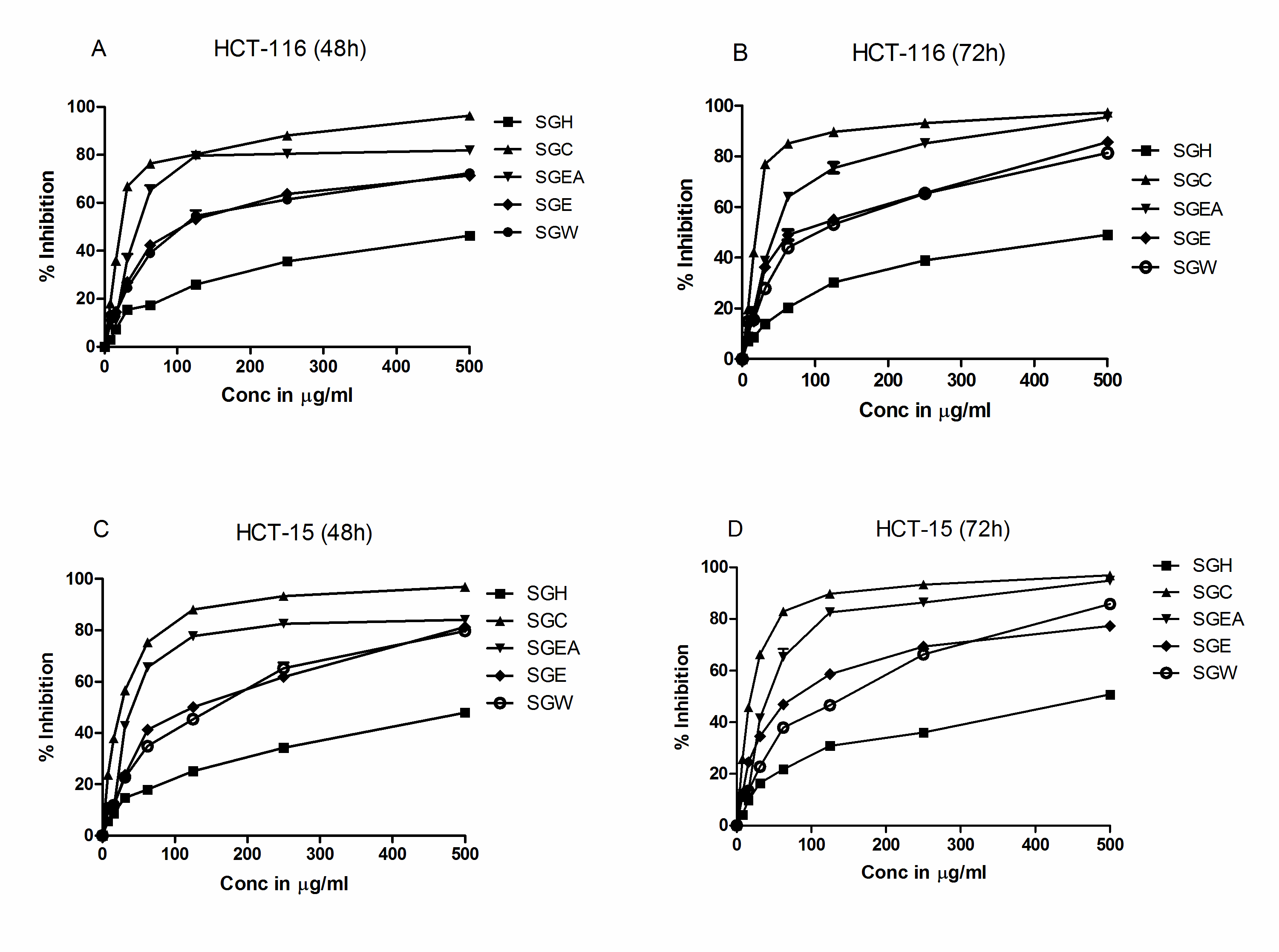


**Supplementary Figure 1:** Extracts of *Simarouba glauca* leaves inhibit colorectal carcinoma cell lines HCT-116 (A and B) and HCT-15 (C and D) at 48 and 72h

**
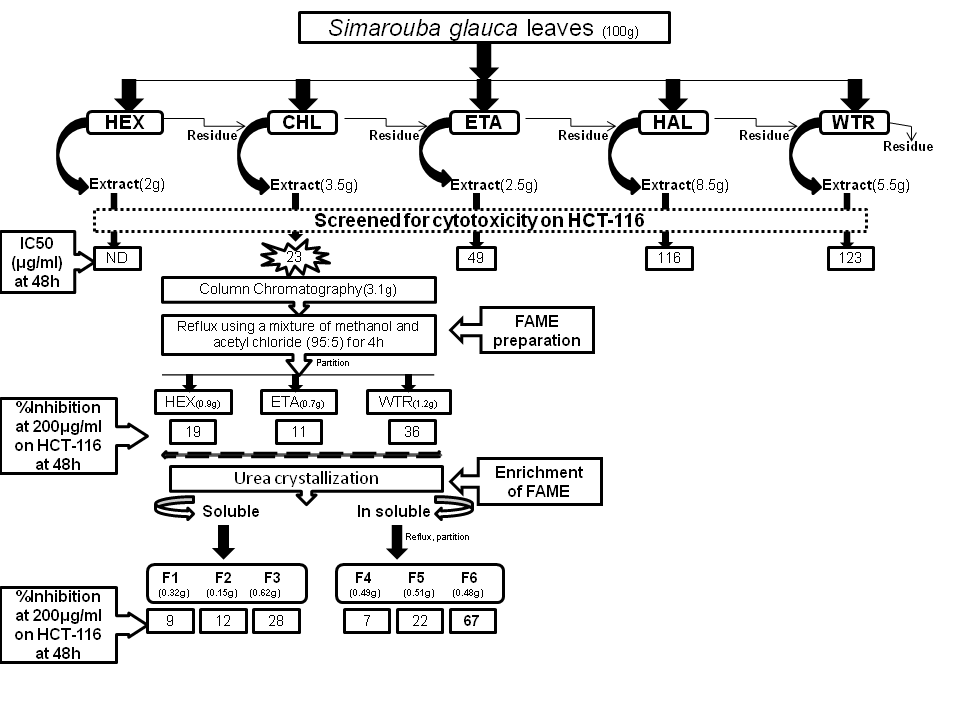
**

**Supplementary Figure 2: Schematic representation of the fractionation procedure developed for isolating Tricaproin rich material:** Leaves of SG were dried and extracted with solvents of increasing hydrophilicity. The extracts were screened for anti-cancer activity and further fractionated using silica gel chromatography. The obtained fractions were tested for anticancer activity and, a fraction rich in tricaproin purified as detailed in materials and methods.


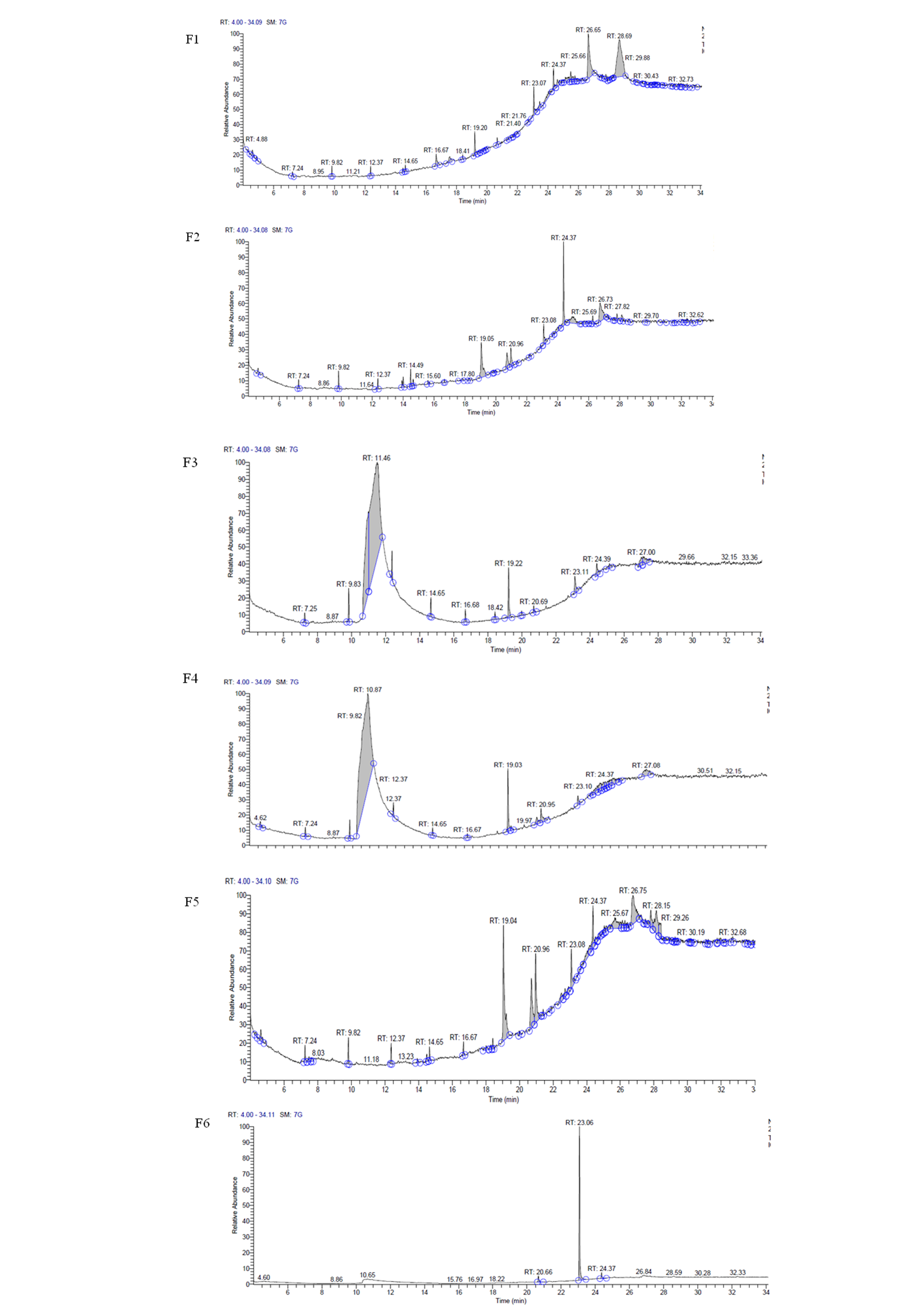


**Supplementary Figure 3:** Analysis of fractions 1-6 using GC-MS


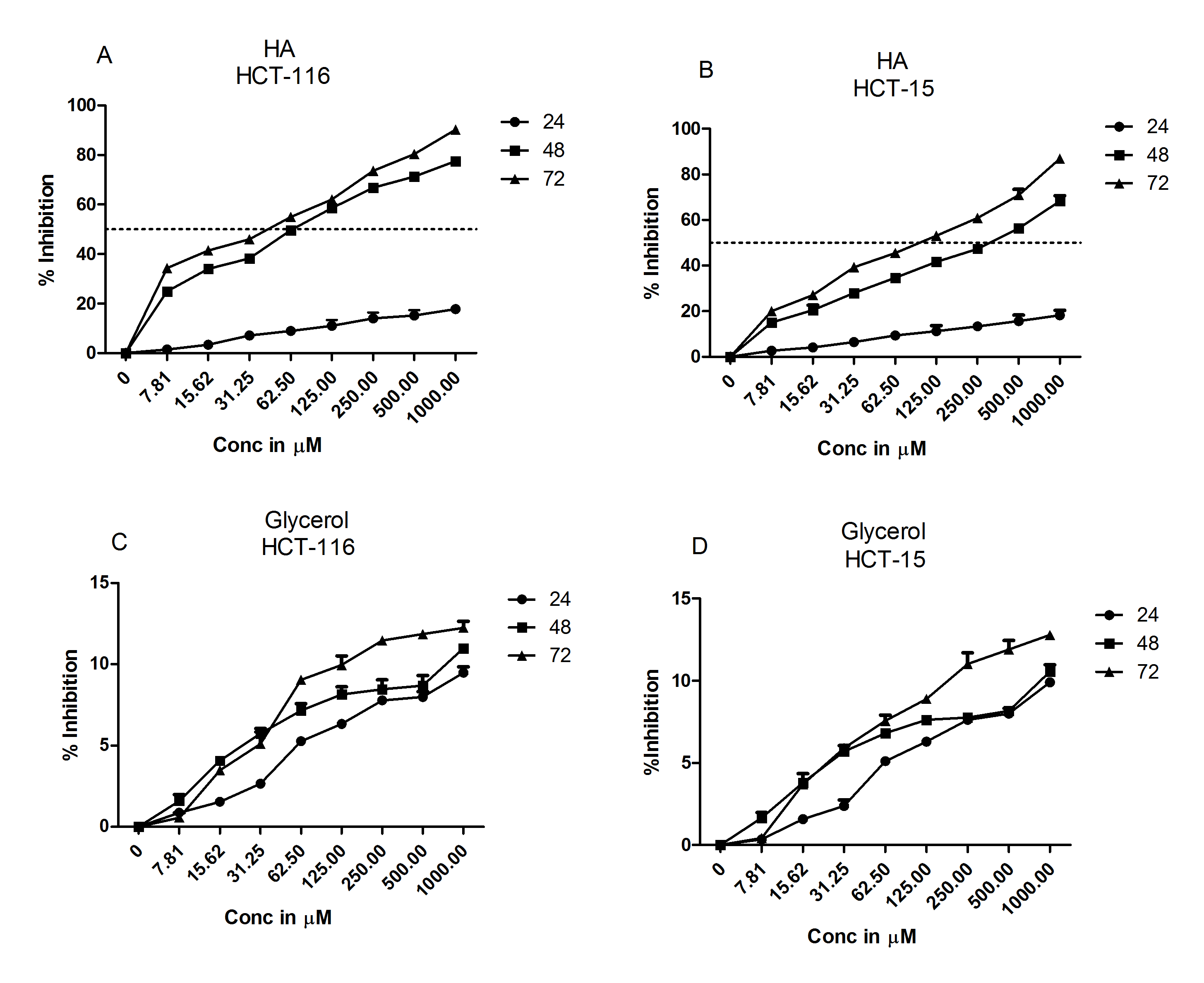


**Supplementary Figure 4**: Hexanoic acid (A and B) but not glycerol (C and D) is more effective at inhibiting colorectal cancer cells HCT-116 (A and C) and HCT-15 (B and D) growth


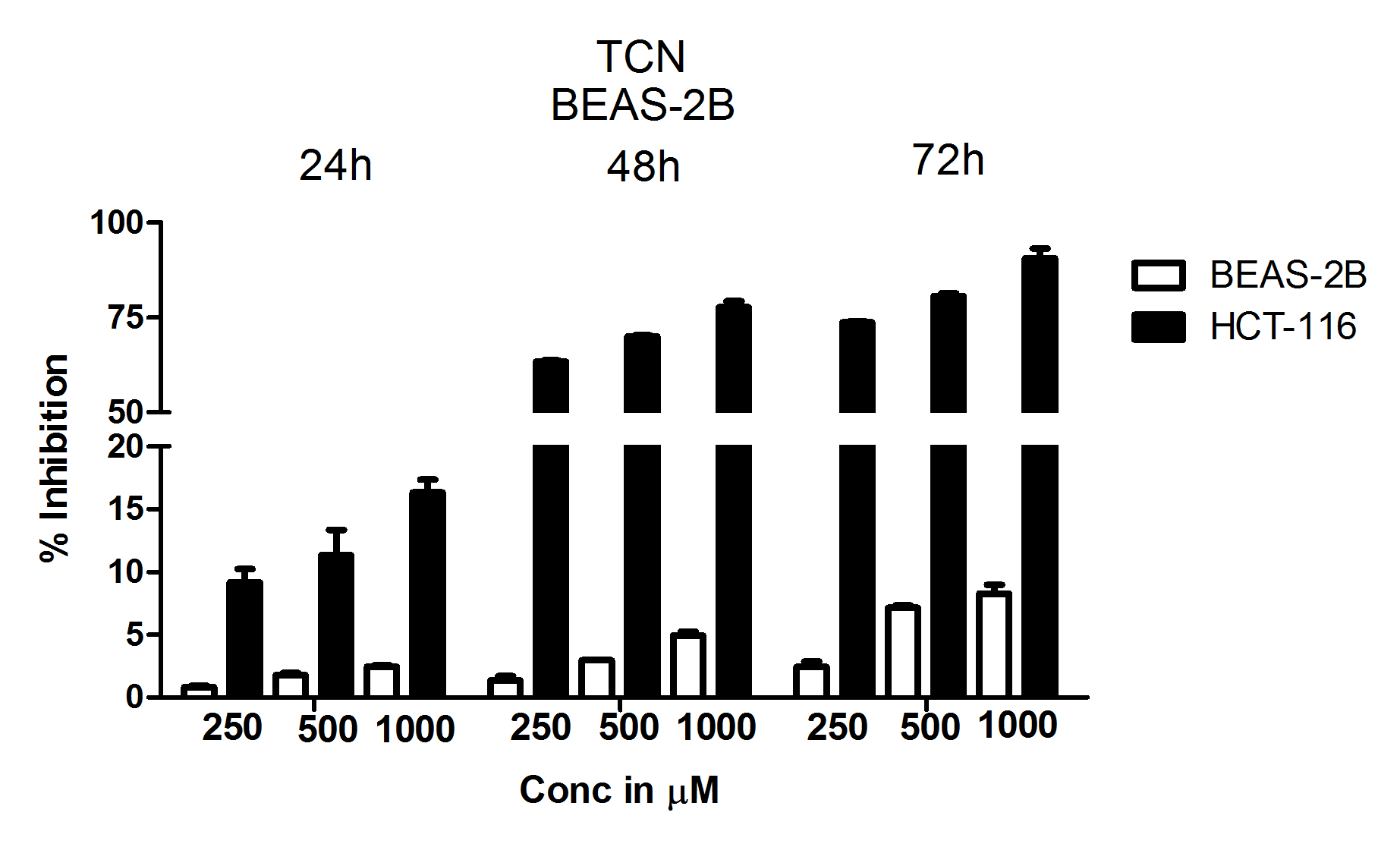


**Supplementary Figure 5: Tricaproin retards the growth of cancer cells but not normal lung epithelial cells:** To check whether isolated tricaproin exhibits similar selectivity as the parent chloroform extract, increasing concentrations of TCN was added to exponentially growing colorectal carcinoma cell line HCT-116 and normal lung epithelial cell line BEAS-2B. Number of viable cells was determined using SRB at 24h, 48h and 72h treatment. TCN inhibit colorectal cancer cells growth with better selectivity to cancer cells.


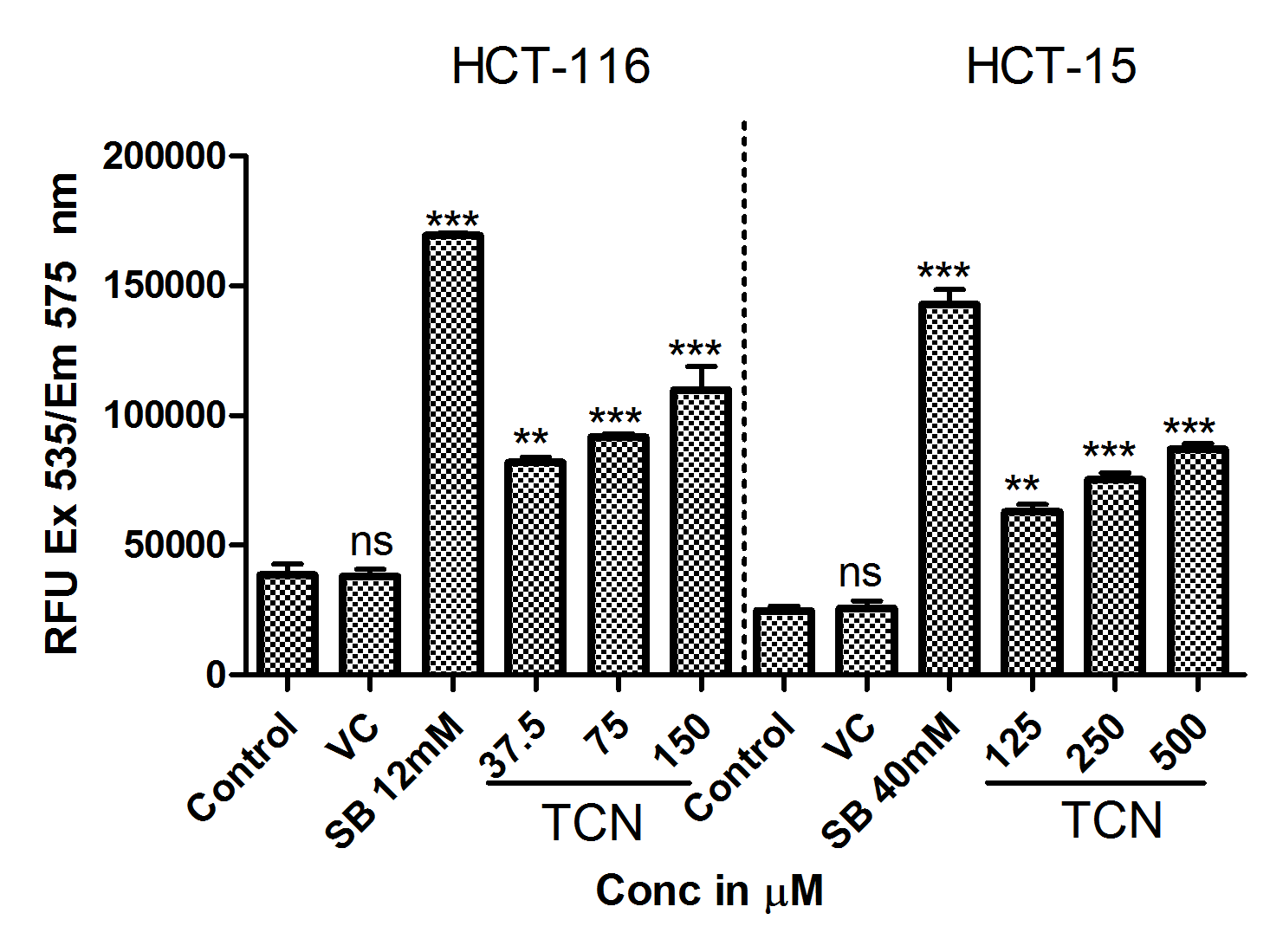


**Supplementary Figure 6:** TCN and positive control sodium butyrate triggers the release of lactate dehydrogenase (an indication of cell death) in to the medium.


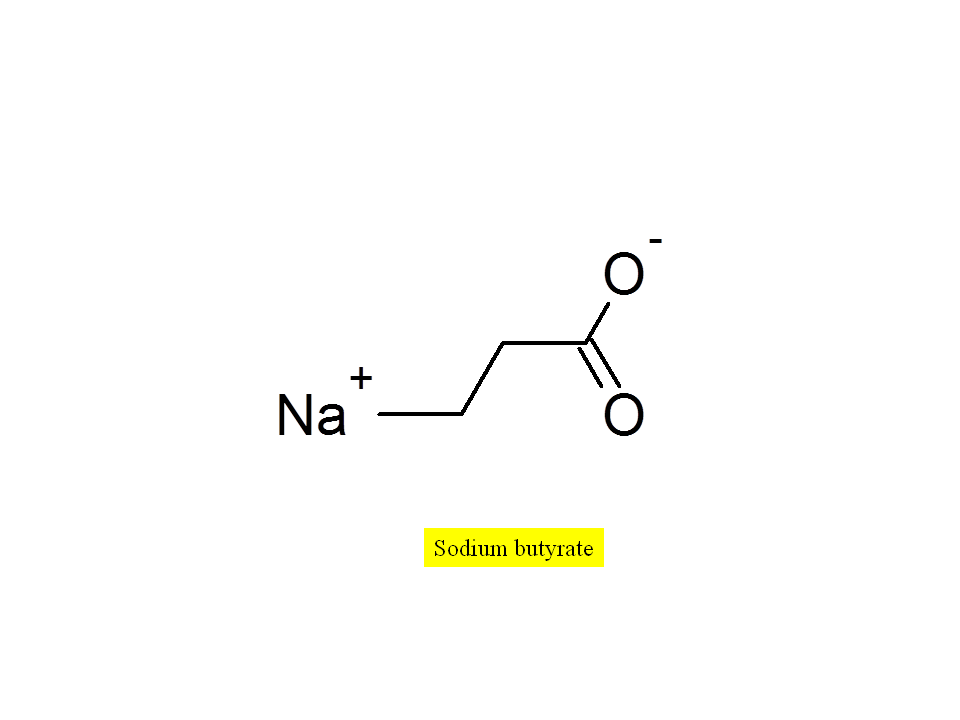


**Supplementary Figure 7: Structure of sodium butyrate:** Sodium butyrate is a short chain fatty acid produced predominantly in large colon. Although a weak inhibitor, it targets HDAC thereby retard the proliferation of cancer cells


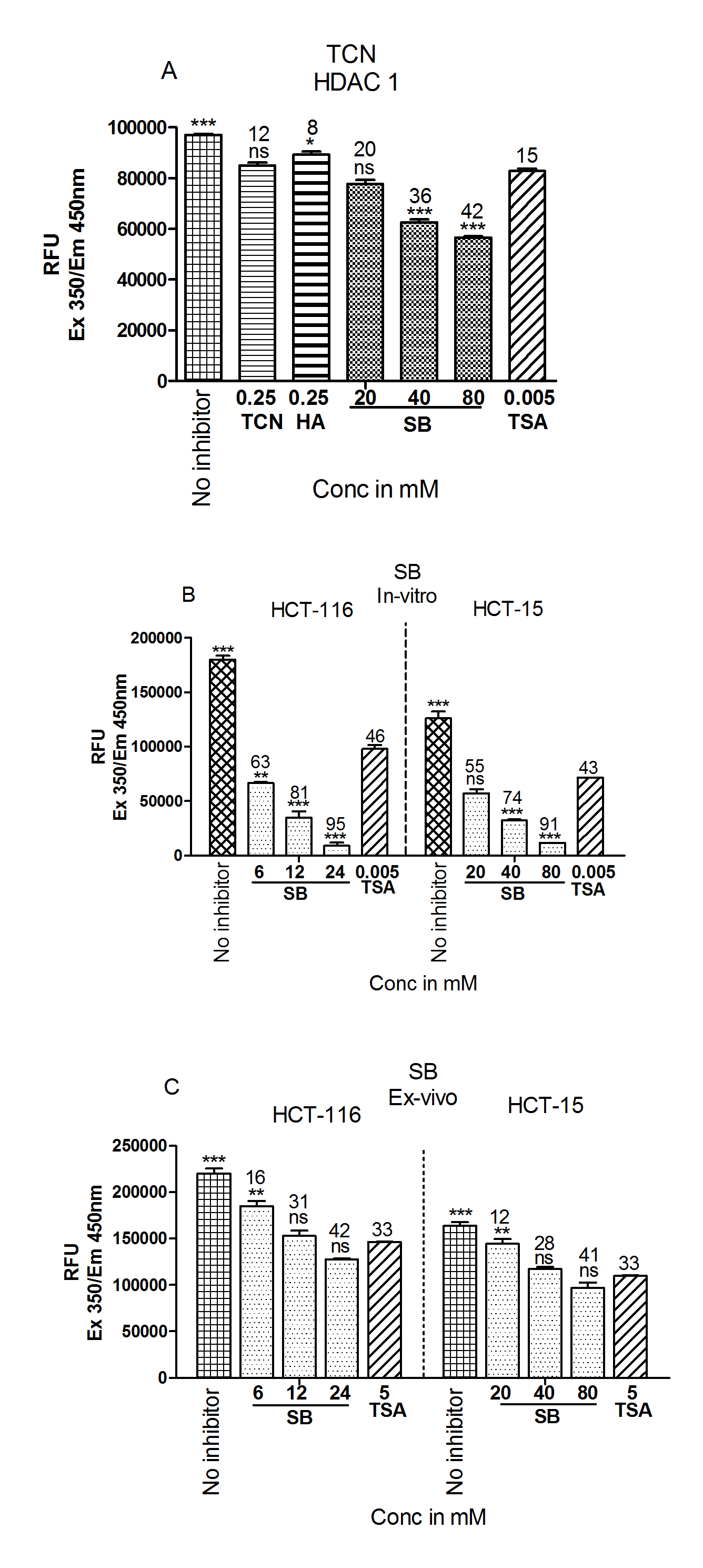


**Supplementary Figure 8:** TCN and HA treatments inhibits HDACs 12% and 8% respectively(A). Positive controls TSA and SB also exhibited similar reduction in percentage HDAC activity inhibition when tested using isolated nuclear extract or commercial HDAC1(B & C)
